# Adeno-associated virus-binding antibodies detected in cats living in the Northeastern United States lack neutralizing activity

**DOI:** 10.1038/s41598-020-66596-4

**Published:** 2020-06-22

**Authors:** Kei Adachi, Gregory A. Dissen, Alejandro Lomniczi, Qing Xie, Sergio R. Ojeda, Hiroyuki Nakai

**Affiliations:** 1 Department of Molecular & Medical Genetics, Oregon Health & Science University School of Medicine, Portland, Oregon 97239 USA; 2 Department of Molecular Microbiology & Immunology, Oregon Health & Science University School of Medicine, Portland, Oregon 97239 USA; 3 Division of Neuroscience, Oregon National Primate Research Center, Beaverton, Oregon, 97006 United States of America; 4 Molecular Virology Core, Oregon National Primate Research Center, Beaverton, Oregon, 97006 United States of America

**Keywords:** Viral vectors, Gene therapy, Gene delivery, Genetic vectors, Biotechnology

## Abstract

Cats are a critical pre-clinical model for studying adeno-associated virus (AAV) vector-mediated gene therapies. A recent study has described the high prevalence of anti-AAV neutralizing antibodies among domestic cats in Switzerland. However, our knowledge of pre-existing humoral immunity against various AAV serotypes in cats is still limited. Here, we show that, although antibodies binding known AAV serotypes (AAV1 to AAV11) are prevalent in cats living in the Northeastern United States, these antibodies do not necessarily neutralize AAV infectivity. We analyzed sera from 35 client-owned, 20 feral, and 30 specific pathogen-free (SPF) cats for pre-existing AAV-binding antibodies against the 11 serotypes. Antibody prevalence was 7 to 90% with an overall median of 50%. The AAV-binding antibodies showed broad reactivities with other serotypes. Of 44 selected antibodies binding AAV2, AAV6 or AAV9, none exhibited appreciable neutralizing activities. Instead, AAV6 or AAV9-binding antibodies showed a transduction-enhancing effect. AAV6-binding antibodies were highly prevalent in SPF cats (83%), but this was primarily due to cross-reactivity with preventive vaccine-induced anti-feline panleukopenia virus antibodies. These results indicate that prevalent pre-existing immunity in cats is not necessarily inhibitory to AAV and highlight a substantial difference in the nature of AAV-binding antibodies in cats living in geographically different regions.

## Introduction

Adeno-associated viral (AAV) vectors are a safe and the most effective *in vivo* gene delivery vehicle for gene therapy. Clinical trials of AAV vector-mediated human gene therapy have shown remarkable success, leading to three commercial products with regulatory approval in western countries^1^. AAV vector-based therapeutic approaches continue to rapidly evolve alongside genome editing technologies^2,3^ and have enormous potential for the treatment of various intractable diseases, far beyond a limited number of rare genetic disorders. Further development of AAV-mediated gene therapy to treat a broader spectrum of diseases requires an appropriate choice of animal models in preclinical studies.

In this regard, feline models offer various advantages over other animal models. Compared to rodent models, cats offer greater similarities to humans in anatomy, physiology and genetics. Compared to larger animals including non-human primates, cats are more tractable due to their size and availability. To date, AAV vector-mediated gene therapy has proven efficacious for feline models of lysosomal storage diseases^4–8^, lipoprotein lipase deficiency^9^ and spinal muscular atrophy^10^. Importantly, AAV vectors can also be used to modify physiological processes such as fertility, thus providing a powerful tool for the control of rapidly expanding feral populations. In this context, AAV vectors have been postulated to serve as a potential one-time non-surgical contraceptive agent that permanently sterilizes cats and dogs for population control^11–13^. Several vectored contraception approaches have been investigated, which include AAV vector-mediated RNA interference (RNAi) in hypothalamic neurons and AAV vector-mediated persisted supplementation of a monoclonal antibody (moAb) into the blood circulation that binds gonadotropin-releasing hormone (GnRH) or the zona pellucida (ZP) surrounding the oocyte. These RNAi and moAb approaches are expected to permanently disrupt the reproductive axis in treated animals. Thus, there is a growing need to understand the fundamental AAV vector biology and virus-host interactions in the context of cats; however, our current knowledge and experiences with feline studies using AAV vectors remain severely limited.

Antigen-specific humoral immunity constitutes the fundamental host defense mechanism that blocks viral entry into the body. Even low titers of pre-existing neutralizing antibodies (NAb) against AAV capsids are sufficient to completely abolish *in vivo* transduction with AAV vectors in mice and non-human primates^14–16^. Cats are naturally infected with parvoviruses such as feline panleukopenia virus (FPV) and canine parvovirus (CPV)^17^, and antibodies against these parvoviruses can cross-react with closely related viruses. For example, preventive vaccine-induced anti-FPV NAbs in cats are capable of protecting against CPV infection^18,19^. These antibodies may also cross-react with AAVs. There have been no known AAVs derived from cats as the primary host; however, it remains possible that primate AAV serotypes have prevalently infected cat populations. Indeed, a recent study of 230 domestic cats living in Switzerland reported that approximately a half of this population had NAbs to one or more of the following AAV serotypes: AAV1, AAV2, AAV5, AAV6, AAV7, AAV8 and AAV9^20^. One potential interpretation of this study is that natural infection with AAVs has induced broadly cross-reacting NAbs to multiple serotypes^21^ in cats living in a particular geographic region. Thus, it is important to address the question as to whether or not cats receiving AAV vectors in experimental and therapeutic settings have pre-existing anti-AAV NAbs. To date, this issue has been largely ignored based on the incorrect assumption that pre-existing anti-AAV NAbs are likely not present in non-primate animals^20,22^.

In this study, we investigated the possibility that cats could carry pre-existing anti-AAV NAbs, thus compromising the gene therapy efficacy of AAV-based vectors. To this end, we performed a set of experiments to investigate the prevalence of naturally occurring antibodies that bind a panel of AAV serotypes (AAV1, AAV2, AAV3, AAV4, AAV5, AAV6, AAV7, AAV8, AAV9, AAV10 and AAV11) in a total of 85 cats living in the Northeastern United States (*i.e*.; 35 client-owned, 20 feral, and 30 specific pathogen-free (SPF) cats). We used an enzyme-linked immunosorbent assay (ELISA) and quantified feline Immunoglobulin G (IgG) that specifically binds each AAV serotype capsid. The neutralizing ability of AAV-binding antibodies was determined using a cell-based anti-AAV NAb assay. These analyses revealed that many cats harbored AAV-binding antibodies for multiple AAV serotypes at varying frequencies. However, unlike the observation of anti-AAV NAbs in cats from Switzerland^20^, all cat serum samples selected from among those showing the highest levels of AAV2, AAV6 or AAV9-binding antibodies showed no appreciable neutralizing activities. Interestingly, cat sera containing a high quantity of AAV6 or AAV9-binding antibody showed instead a transduction-enhancing effect, similar to human sera containing non-neutralizing AAV8 binding antibodies^23^. These observations indicate that pre-existing anti-AAV humoral immunity in cat populations in the United States will not necessarily have a negative impact on AAV vector transduction. In addition to this finding, we discovered a potential mechanism leading to the presence of non-neutralizing AAV6-binding antibodies in SPF cats and identified a potential epitope recognized by these antibodies on the AAV6 capsid.

## Results

### Experimental design

The aim of this study was to determine the prevalence of pre-existing antibodies against a panel of naturally occurring AAV serotypes (AAV1 to AAV11) in cats and their ability to neutralize AAV infection. For the AAV neutralizing assay, we selected the following three serotypes: AAV2, AAV6 and AAV9. AAV2 was chosen because it serves as the prototype of AAV and has been most extensively studied; AAV9 was chosen because of its ability to robustly transduce a variety of therapeutically-relevant organs *in vivo* and its potential as an attractive platform for capsid engineering^24^. AAV6 was selected because we found a subgroup of cats exhibiting a high prevalence of AAV6-binding antibodies (see below). We obtained cat sera from three different sources that represent samples of cat populations living in the Northeastern United States (Table 1). A total of 99 cats split into four different groups were included in the study: Group 1, client-owned cats (n = 35, gender and ages, not reported); Group 2, feral cats (n = 20; gender and ages, not reported); Group 3, specific pathogen-free (SPF) cats (n = 30; 15 males and 15 females at ages of 4 to 10 months); and Group 4, a cohort of 14 SPF cats (7 each of males and females at the age of 5 months) whose birthdates fall within a date range of 5 days. All cats in Group 3 had been vaccinated against feline panleukopenia virus (FPV) with Fel-O-Vax (Boehringer Ingelheim, Ridgefield, CT) one to three times at least 8 days before the serum samples were collected. Both pre and post-vaccinated (pre-vac and post-vac) samples were collected from all animals in Group 4. Detailed information about the cats and their serum samples can be found in Supplementary Table S1.

**Table 1:** A summary of the cats.

Group	Category	No. of cats	Source	Residential area of cats	FPV vaccine status	Notes
1	Client-owned	35	Animal Health Diagnostic Center (Cornell University)	Mostly in NY	Unknown	Cat strains, not reported.
2	Feral	20	Biochemed Services	PA	Unknown	Cat strains, not reported.
3	SPF	30	Liberty Research Inc.	NY	Post-vaccinated	DSH cats.
4	SPF	14	Liberty Research Inc.	NY	Pre and post-vaccinated	DSH cats. Used for a cohort study.

NY, New York; PA, Pennsylvania; FPV, feline panleukopenia virus, DSH, domestic short-haired.

### Absence of antibodies against AAV1 to AAV11 in the majority of SPF cats before FPV vaccination

**Figure 1:**
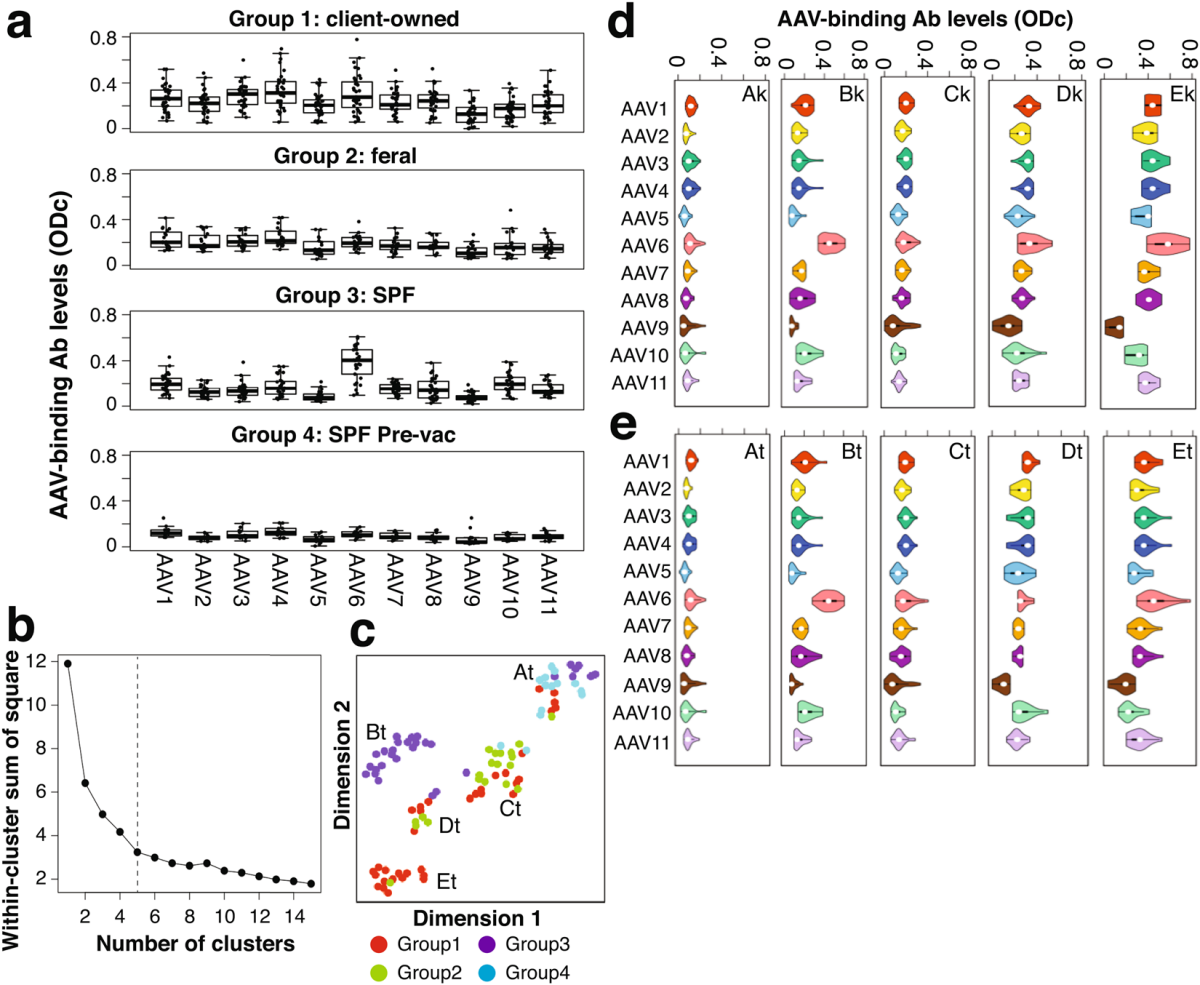
Prevalence of AAV-binding antibodies in sera collected from four different cat populations comprising a total of 99 cats living in the Northeastern United States. (**a**) Box plots showing levels of IgG antibodies in cat serum samples that bind to each AAV serotype capsid in each group: Group 1, 35 client-owned cats; Group 2, 20 feral cats; Group 3, 30 SPF cats that had received FPV vaccine; and Group 4 Pre-vac, 14 SPF cats that had not received FPV vaccine. The quantities of AAV-binding IgG antibodies were determined by AAV-binding antibody ELISA for each serotype using 1:5-diluted serum samples and expressed as ODc. The values indicate averages of technical duplicates. **(b)** Selection of an optimal number of clusters used for a k-means clustering analysis of a total of 99 cats. The graph shows within-cluster sum of squares (WSS) values as a function of the number of clusters (k). We selected an elbow point at k = 5, indicated with a dotted line, as an optimal k for clustering. The k-means clustering identified 5 clusters, Clusters Ak to Ek (Supplementary Table S1). **(c**) A t-SNE plot showing the distribution of cats from 4 different populations (Group 1, red; Group 2, green; Group 3, purple; and Group 4 Pre-vac, cyan). They form 5 distinct clusters (Clusters Ak to Ek, Supplementary Table S1) based on the AAV-binding antibody spectra. **(d**,**e)** Violin plots showing AAV-binding antibody levels for each serotype in each cluster identified by k-means clustering (**d**) and visualized by t-SNE (**e**).

We determined the levels of humoral immunity against AAV1 to AAV11 in cats by an ELISA that quantifies feline IgG antibodies binding intact AAV virions of each serotype. In this measurement, we expressed each AAV serotype-specific IgG antibody level as a corrected OD value (ODc). This analysis revealed that SPF cats without vaccination (Group 4 Pre-vac animals) exhibited lower AAV binding antibody levels than the other three groups (Fig. 1a). Since no cats, including SPF cats, had been known *a priori* to be bona-fide negative for AAV-binding antibodies, we first sought to identify *a posteriori* AAV-binding antibody-negative cats, which should form a group distinct from antibody-positive cats, by using unsupervised methods. To this end, we employed k-means non-hierarchical clustering and t-distributed stochastic neighbor embedding (t-SNE). We selected k = 5 as an optimal number of k-means clustering by calculating the total within-cluster sum of square (WSS) values (Fig. 1b). Dimensionality reduction by t-SNE also allowed us to visualize 5 clusters of cat populations (Fig. 1c). The five clusters identified by k-means clustering (Clusters Ak to Ek) and those visualized by t-SNE (Clusters At to Et) have significant overlaps (Supplementary Table S2). Cluster A (both Ak and At) presenting cats with very low ODc values for all AAV serotypes could be unambiguously segregated from the other four clusters showing medium to high ODc values for multiple serotypes (Fig. 1d,e). Among the 14 Group 4 Pre-vac cats, 13 and 12 cats are in Clusters Ak and At, respectively. These Group 4 Pre-vac cats had been maintained under an SPF condition, were all young (5 months old), and had not been vaccinated, having least chances of viral infection. In addition, maternally derived passive immunity should be substantially attenuated by the age of 5 months. These observations strongly support the conclusion that the majority of Group 4 Pre-vac cats are negative for pre-existing immunity against multiple AAV serotypes. Thus, we defined cut-off ODc values in the ELISA using these cats to determine whether cats are positive or negative for AAV-binding antibodies for each serotype. This ELISA revealed that Group 4 Pre-vac cats were seronegative for the majority of AAV serotypes. Only 1 to 3 cats out of 14 (7 to 21%) were seropositive for AAV1, AAV8, AAV9 and AAV11 but they did not carry antibodies that react with many different AAV serotypes (Table 2). Although 6 out of 14 (43%) were found to be positive for 1 or 2 serotypes, their antibody levels were all low.

**Table 2:** Seroprevalence of anti-AAV humoral immunity in cats in the Northeastern United States.

Group	AAV serotypes											
	AAV1	AAV2	AAV3	AAV4	AAV5	AAV6	AAV7	AAV8	AAV9	AAV10	AAV11	Any
G1	60	77	71	60	63	74	63	83	54	49	69	91
G2	40	90	35	35	35	50	35	90	50	30	40	90
G3	33	33	10	20	7	83	20	63	20	53	37	100
G4 Pre-vac	7	0	0	0	0	0	0	21	14	0	7	43
G4 Post-vac	n.d.	n.d.	n.d.	n.d.	n.d.	n.d.	n.d.	n.d.	n.d.	n.d.	n.d.	n.d
**Group**	**No. of AAV serotypes exhibiting seropositive in the same animal**											
	**None**	**1**	**2**	**3**	**4**	**5**	**6**	**7**	**8**	**9**	**10**	**All**
G1	9	9	0	3	9	9	0	3	3	6	31	20
G2	0	5	20	20	10	5	10	5	0	0	10	15
G3	10	13	17	17	3	13	10	3	7	3	0	3
G4 Pre-vac	57	36	7	0	0	0	0	0	0	0	0	0
G4 Post-vac	n.a.	n.a.	n.a.	n.a.	n.a.	n.a.	n.a.	n.a.	n.a.	n.a.	n.a.	n.a.

The values are percentage of cats seropositive for each AAV serotype (the upper half) or a combination of AAV serotypes (the bottom half) in each group. Group 1 (G1), 35 client-owned cats; Group 2 (G2), 20 feral cats; Group 3 (G3), 30 SPF cats that received an FPV vaccine; and Group 4 (G4), a cohort of 14 SPF cats before vaccination (G4 Pre-vac) and after vaccination (G4 Post-vac). Please note that the antibodies we assayed here include both non-neutralizing antibodies and NAbs. n.d., not done; n.a., not applicable.

### High prevalence of broadly reacting AAV-binding antibodies in client-owned, feral and post-vaccinated SPF cats

**Figure 2:**
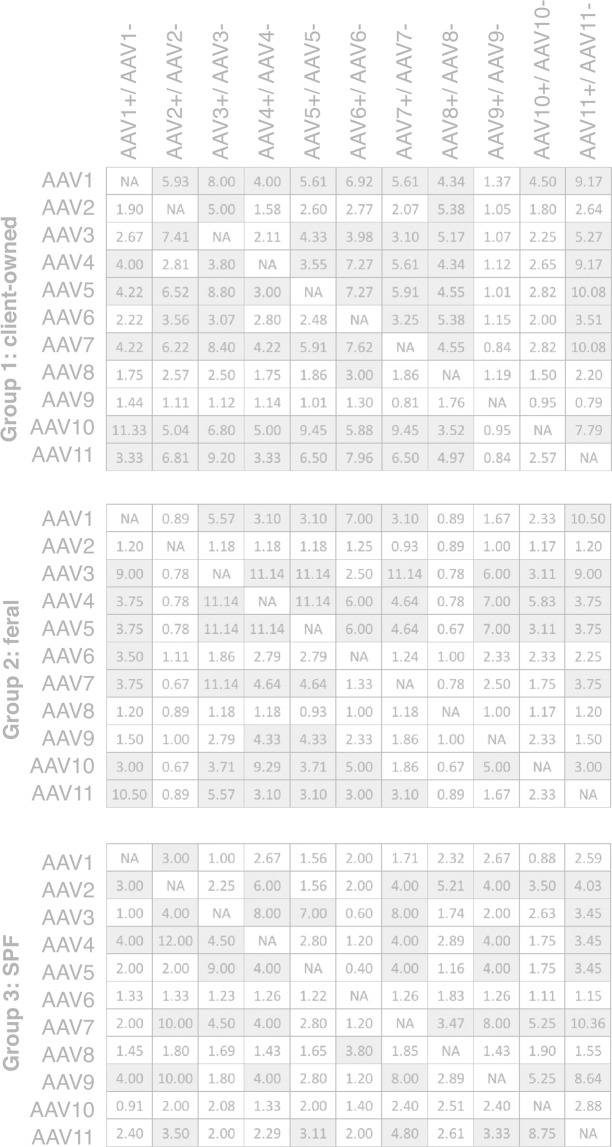
Seroreactivity linkage analysis between two different AAV serotypes. A seroreactivity linkage analysis^25^ was performed in each study group (Groups 1, 2 and 3) using the binary seropositivity data. The numbers indicate positive (>1) and negative (<1) linkages. For example, the value of 1.90 at the crossing point of AAV2 and AAV1+/AAV1− in Group 1 indicates that AAV2-binding antibody positivity in AAV1-binding antibody-positive animals is 1.90-times higher than AAV2-binding antibody positivity in AAV1-binding antibody-negative animals. The serotype combinations showing the linkage values of >3 are gray-shadowed.

Unlike the Group 4 Pre-vac cats, many of the Group 1 (client-owned) and Group 2 (feral) cats and Group 3 (post-vaccinated SPF) cats had AAV-binding antibodies that are reactive with many different serotypes to a varying extent (Table 2). Antibody prevalence in these three groups was 7 to 90%, depending on the AAV serotype, with an overall median of 50%. AAV-binding antibodies from Group 1 cats reacted with more AAV serotypes than those in Groups 2 and 3 cats with statistical significance (P = 0.00002 to 0.036, a two-sided Boschloo’s exact unconditional test followed by Bonferroni correction) (Supplementary Tables S3–S5). There was no statistically significant difference in the extent of the broad reactivity between Groups 2 and 3. High prevalence of broadly reacting AAV-binding antibodies was also demonstrated by a seroreactivity linkage analysis reported by Calcedo *et al*.^25^ (Fig. 2). In a three-way comparison of the number of serotype-serotype combinations that exhibit a linkage value of more than 3 (*i.e*., more than a modest linkage^25^) between Groups 1, 2 and 3, a comparison between Groups 1 and 3 showed a statistically significant difference. That is, the antibody reactivity linkage in Group 1 was greater than that in Group 3 (P = 0.0012, a two-sided Boschloo’s exact unconditional test followed by Bonferroni correction) although the other two comparisons yielded no significant difference (Supplementary Table S6). It should be noted that, although AAV-binding antibodies in Group 3 SPF cats showed broad reactivity, the prevalence and levels of AAV-binding antibodies were lower than those of Groups 1 and 2 cats (Fig. 1a and Supplementary Table S7). It should also be noted that the observed broad reactivity of polyclonal AAV-binding antibodies present in cats’ sera does not necessarily indicate that each moAb can cross-react with multiple AAV serotypes. It still remains possible that an exposure to one AAV serotype greatly increases the risk of exposure to many other AAV serotypes, producing broadly reacting polyclonal AAV-binding antibodies. Such an exposure to multiple AAV serotypes could occur in an environment where humans, the primary host of many AAV serotypes, and cats have intensive interactions. Nonetheless, AAV-binding antibodies developed in both non-SPF cats and SPF cats can broadly react with multiple AAV serotypes with the degree of broad reactivity being highest in client-owned cats, lowest in post-vaccinated SPF cats, and intermediate in feral cats.

### Post-vaccinated SPF cats showed distinctively high levels of AAV6-binding IgG antibodies

Intriguingly, the AAV-binding IgG antibody ELISA on cat serum samples revealed that Group 3 cats showed an AAV-binding antibody spectrum distinct from other groups in that the prevalence and the levels of AAV6-binding antibodies were found exclusively high among all the 11 serotypes (Table 2 and Fig. 1). The frequency of seropositivity against AAV6 was significantly higher than that of other serotypes (P = 2.2 × 10^−8^ to 0.011, a two-sided Boschloo’s exact unconditional test followed by Bonferroni correction) with the exceptions of AAV8 (P = 1.0) and AAV10 (P = 0.75) (Supplementary Table S8). The lack of statistical significance for AAV8 and AAV10 is most likely due to the binary nature of the seropositivity assessment which allows cats with high antibody levels and those with low levels to belong to the same seropositive category. Statistical assessment using AAV-binding antibody levels revealed that AAV6-binding antibody levels were significantly higher than the levels of any other serotypes in Group 3 (P = 1.0 × 10^−7^ to 1.1 × 10^−5^, a two-sided Wilcoxon signed-ranked test followed by Bonferroni correction) (Supplementary Table S8). The AAV6-binding antibodies in Group 3 were also significantly higher than those in Groups 1 and 2 (P = 2.0 × 10^−5^ to 9.2 × 10^−3^, a two-sided Mann-Whitney U-test followed by Bonferroni correction) (Supplementary Table S7). This AAV-binding antibody spectrum observed in the Group 3 SPF cats that had received FPV vaccination cats was in stark contrast to that observed in the Group 4 Pre-vac SPF cats that had not received FPV vaccination (Fig. 1a).

### Induction of AAV6-binding IgG antibodies by FPV vaccination

**Figure 3:**
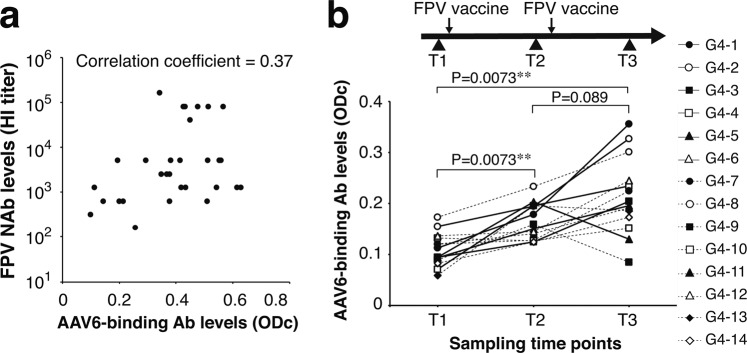
Cross-reactivity of FPV antibodies with AAV6. (**a**) Correlation between anti-FPV NAb titers and AAV6-binding antibody levels in Group 3. All 30 Group 3 SPF cats had received FPV vaccine one to three times before sample collection. The anti-FPV NAb titers (Y-axis) and AAV6-binding antibody levels (X-axis) were determined by a hemagglutination inhibition (HI) assay and an AAV6-binding antibody ELISA, respectively. Each dot represents one animal. Spearman’s rank correlation coefficient = 0.37, P = 0.043. **(b**) AAV6-binding antibody levels before and after FPV vaccination in a cohort of 14 SPF cats. AAV6-binding antibodies levels were monitored three times (T1, T2 and T3) in a 36-day period. T1, Day 0; the first FPV vaccine administration, Day 1; T2, Day 20; the second FPV vaccine administration, Day 22; and T3, Day 36. A two-sided Wilcoxon signed-ranked test followed by Bonferroni correction was used to determine P values. Asterisks indicate statistical significance (**P < 0.01).

FPV is a ubiquitous and highly contagious parvovirus that causes feline enteritis and leukopenia leading to high mortality^17^. Thus, preventive vaccination to FPV in cats is a common veterinary practice in veterinary medicine in the United States^26^. All Group 3 cats, which had received FPV vaccine, were positive for anti-FPV NAbs showing hemagglutination inhibition (HI) titers ranging between 1:160 and 1:163,840 serum dilutions with a median of 1:5120. There was a statistically significant positive correlation between anti-FPV NAb titers and AAV6-binding antibody levels in Group 3 cats (P = 0.043, Spearman’s rank correlation coefficient = 0.37) (Fig. 3a). With this positive correlation and the fact that FPV and AAV are close neighbors both phylogenetically and structurally, we hypothesized that FPV vaccination could raise antibodies that cross-react with AAV6. To address this hypothesis, we followed up AAV6-binding antibody levels in the Group 4 cohort before and after FPV vaccination. We observed a statistically significant increase in AAV6-binding antibody levels within a 36-day observation period consisting of: Day 0, pre-vaccination serum sampling (T1); Day 1, first vaccination; Day 20, second serum sampling (T2); Day 22, a booster injection; and Day 36, the third serum sampling (T3). (Fig. 3b). The prominence of AAV6-binding antibodies was also apparent in the Cluster E cats identified by t-SNE dimensionality reduction (Fig. 1e). In Cluster E cats, AAV6-binding antibody levels were significantly higher than all other serotypes except for AAV1, AAV3 and AAV4 (P = 1.8 × 10^−7^ to 0.030, a two-sided Wilcoxon signed-ranked test followed by Bonferroni correction). Notably, AAV6 is the only serotype that achieved significantly higher antibody levels compared to the levels of any other serotype (P < 0.05). This peculiar AAV-binding antibody spectrum showing a unique predominance of AAV6-binding antibodies is reminiscent of the antibody profile of the Group 3 SPF cats having received FPV vaccine. Remarkably, Cluster E cats almost exclusively belonged to the Group 1 client-owned cats (13 of 14, 93%), with only one animal in this cluster belonging to the Group 2 feral cats. Indeed, the Group 1 client-owned cats developed the peculiar AAV-binding antibody profile with an AAV6 antibody predominance much more frequently (13/35, 37%) than the Group 2 feral cats (1/20, 5%) (P = 0.0078, a two-sided Boschloo’s exact unconditional test). The majority of the Group 1 client-owned cats likely had received FPV vaccine in light of the Canine and Feline Preventive Healthcare Guidelines introduced by the American Veterinary Medical Association (AVMA) and American Animal Hospital Association (AAHA)^26^ while the majority of the Group 2 feral cats likely did not. Taken altogether, we conclude that FPV vaccination raises antibodies that cross-react exclusively with AAV6 among the 11 serotypes tested.

### Lack of neutralizing ability in AAV2, AAV6 and AAV9-binding antibodies

**Figure 4:**
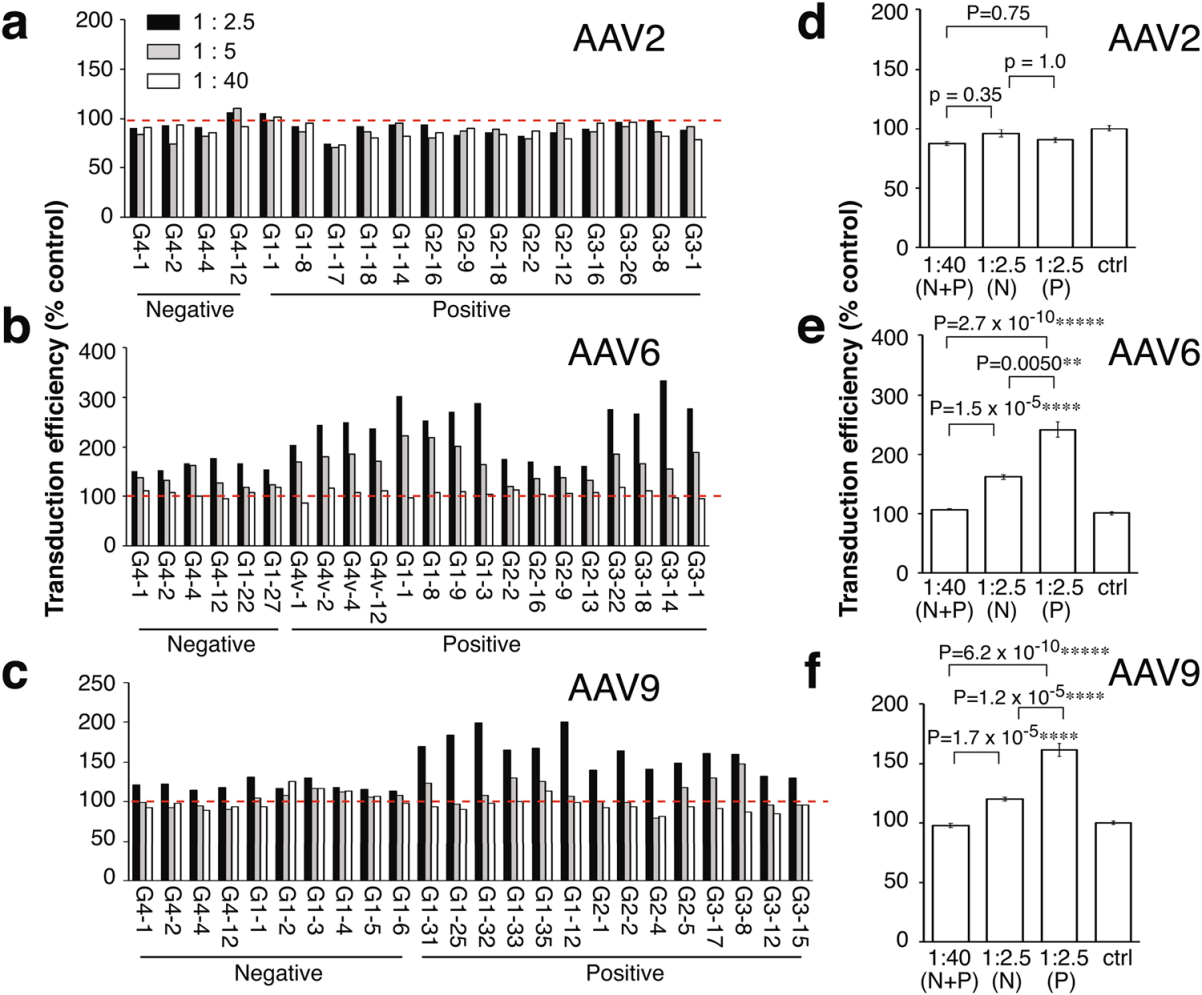
Anti-AAV2, AAV6 and AAV9-NAb assays. (**a**) *In vitro* anti-AAV2 NAb assay was performed as described in Methods to investigate the presence or absence of anti-AAV2 NAbs in cat sera. The Y-axis shows transduction efficiency of reporter cells with AAV2 vector pre-incubated with sample sera, relative to a non-serum condition. Serum samples were diluted at 1:2.5, 1:5 and 1:40 and used for the assay. Results of individual animals are shown with animal identification numbers (IDs). ID Gx-y indicates x = Group number and y = animal number specific to each animal in each group. G4 and G4v indicates Group 4 Pre-vac and Post-vac animals, respectively. A red dotted line indicates the 100% level. **(b**,**c)** The same assay was performed for anti-AAV6 NAbs (**b**) and anti-AAV9 NAbs (**c**) using AAV6 and AAV9 vectors, respectively. **(d–f)** The data shown in Panels a, b and c are combined in bar graphs shown as Panels d, e and f, respectively, for a statistical assessment. In the 1:40 (N + P) group, the results obtained from 1:40-diluted samples in both AAV-binding antibody-positive and negative groups are combined. In the 1:2.5 (N) and 1:2.5 (P) groups, the results obtained from 1:2.5-diluted samples in AAV-binding antibody-positive and negative groups are combined separately, respectively. The controls (ctrl) are references obtained from no serum-containing samples. Error bars indicate mean +/− SEM of biological replicates except for the controls for which we obtained the values from technically duplicated measurements. A two-sided Mann Whitney U-test followed by Bonferroni correction was used to determine P values. Asterisks indicate statistical significance (**P < 0.01. ****P < 0.00001, *****P < 0.000001). For more information about the animal IDs, please refer to Supplementary Table S1.

AAV-binding antibodies do not necessarily harbor the ability to neutralize virus infection^23^. Thus, we conducted an *in vitro* cell-based NAb assay to determine the neutralizing ability of select cat serum samples that were either positive or negative for AAV-binding antibodies. We performed the NAb assay on AAV2, AAV6 and AAV9. AAV2 and AAV9 were selected for the reasons outlined earlier. AAV6 was chosen because our study revealed the high prevalence of AAV6-binding antibodies in the cat populations we analyzed. As predicted, all the cat samples selected from Group 4 Pre-vac had no appreciable NAbs even at the lowest serum dilution we used (1:2.5 dilution), confirming that these cat serum samples represent bona-fide anti-AAV humoral immunity-negative samples (Fig. 4). A total of 14 AAV2-, 16 AAV6-, and 14 AAV9-binding antibody-containing cat sera were analyzed for the presence of NAbs. Surprisingly, none of the AAV-binding antibody-containing cat sera including those carrying high levels of antibodies (Supplementary Fig. S1) showed any inhibitory effects on AAV2, AAV6 or AAV9 transduction in cells even at the lowest serum dilution (1:2.5) (Fig. 4). We acknowledge that these observations may not necessarily be extended to other untested samples or serotypes. However, our observation that none of the 44 tested samples carrying AAV-binding antibodies were positive for NAbs raises the previously unappreciated possibility that a significant fraction of AAV-binding antibodies developed in cats living in particular geographical regions (in the Northeastern United States in our case) could be devoid of neutralizing ability.

### Sera containing AAV6 or 9-binding antibodies enhanced AAV vector transduction

AAV-binding antibodies can mediate transduction-enhancing effects^23^. In our study, we also observed similar effects on reporter cell transduction *in vitro* with AAV6-binding antibodies and AAV9-binding antibodies but not with AAV2-binding antibodies. Pre-incubation of an AAV9 reporter vector (AAV9-CMV-luc) with AAV9-binding antibody-containing cat sera at 1:2.5 dilution enhanced CHO-Lec2 cell transduction by 1.6-fold while this effect was attenuated down to an unappreciable level when 1:40-diluted sera were used (Fig. 4). AAV6-binding antibody-containing sera also enhanced CHO-K1 cell transduction with AAV6-CMV-luc by 2.4-fold. Interestingly cat sera devoid of AAV6-binding antibodies also enhanced transduction by 1.6-fold. All of these enhancements were statistically significant (P = 2.7 × 10^−10^ to 1.5 × 10^−5^, a two-sided Mann-Whitney U-test followed by Bonferroni correction, see Fig. 4**)**. These enhancing effects were not observed with AAV2-binding antibody-containing sera. Taken together, cat sera containing non-neutralizing AAV6 and AAV9 antibodies have the ability to moderately enhance transduction at least in the conditions we employed. However, these observations cannot rule out the possibility that an undefined serum component that emerged together with anti-AAV immunity, other than IgG antibodies detectable by our ELISA, was at least in part responsible for of the observed enhancing effect. In addition, our data reveal that cat serum by itself contains an AAV6 transduction enhancing factor that is likely not associated with anti-AAV immunity because AAV6-binding antibody negative cat sera enhanced reporter cell transduction (Fig. 4**)**.

### An AAV6-binding antibody epitope likely contains K531 in the AAV6 capsid protein

**Figure 5:**
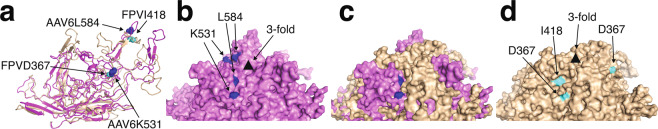
A potential epitope on the AAV6 capsid that cross-reacts with anti-FPV antibodies. (**a**) Structures of AAV6 capsid subunit (magenta) and FPV capsid subunit (wheat) are overlaid using the LSQ algorithm in COOT (https://smb.slac.stanford.edu/facilities/software/coot/#Files)^49,50^ and shown in a ribbon diagram. Blue and cyan space-filling amino acids represent the two pairs of AAV6 and FPV amino acids, respectively, under study (*i.e*., AAV6 L584-FPV I418 shown in the right upper loop and AAV6 K531-FPV D367 near the center). **(b–d)** Side views of surface-rendered images of the three-fold symmetry axis of an AAV6 capsid trimer (**b**), an overlaid AAV6 trimer with FPV trimer aligned with the 3-fold symmetry axis (**c**), and an FPV capsid trimer (**d**). AAV6 and FPV-derived amino acids are indicated with blue and cyan. Local differences in topology is substantially smaller at the AAV6 K531-FPV D367 region than the AAV6 L584-FPV I418 region, indicating that FPV antibody likely recognizes an AAV6 K531-containing local topology as an antibody-binding epitope. The images were generated by PyMOL^51^.

AAV1 and AAV6 VP1 capsid proteins have only 6 amino acid differences; therefore, these two serotype capsids exhibit a high degree of structural similarity^27^. However, our data showed that there is no statistically significant correlation between AAV1-binding antibody levels and AAV6-binding antibody levels in the Group 1 cats (P = 0.078, Spearman’s rank correlation test). This indicates that the main AAV6-binding antibody epitope should reside on the AAV6 capsid surface that include at least one of the 6 amino acids that differ between AAV1 and AAV6. Among these 6 amino acids, F129, D418 and H642 are not exposed on the outer surface of the capsid and V598 is located at the bottom of the center pit at the three-fold symmetry axis, making it unlikely that they are amino acids that interact with antibodies. In contrast, K531 and L584 reside on the outer surface of the AAV6 capsid. Thus, we reasoned that either K531 or L584 is an amino acid able to interact with AAV6-binding antibodies induced by FPV vaccination. A three-dimensional structure alignment of AAV6 and FPV viral capsids identified amino acids D367 and I418 in the FPV capsid VP1 protein as the amino acids corresponding to AAV6 K531 and L584, respectively. We then compared two quaternary structures, one made of AAV6 K531 and its directly neighboring amino acids and another made of FPV D367 and its directly neighboring amino acids, and assessed their structural similarity by both visual inspection (Fig. 5) and determining the root-mean-square deviation (RMSD) in Cα positions (*i.e*., AAV6 K531-FPV D367 comparison) (Supplementary Table S9). We applied the same approach to the AAV6 L584-FPV I418 comparison (Fig. 5 and Supplementary Table S10). Both visual inspection and RMSDs (4.87 Å and 7.37 Å for AAV6 K531-FPV D367 and AAV6 L584-FPV I418 comparisons, respectively) suggested that the AAV6-binding antibody epitope is likely the outer region containing AAV6 K531.

## Discussion

In this study, we determined the prevalence of antibodies binding to the 11 known AAV serotypes in cats living in the Northeastern United States using an ELISA. We also determined the ability of these AAV-binding antibodies to neutralize AAV infectivity using an *in vitro* cell-based NAb assay. Recently, Li *et al*. have reported approximately 50% prevalence of NAbs against AAV1, 2, 6, 7, 8, and 9 in domestic cat populations in Switzerland^20^. Although both studies report that pre-existing humoral immunity against AAV is prevalent in cat populations, we report that these AAV-binding antibodies demonstrate a surprising lack of neutralizing ability. Since we assessed the presence of NAbs in a subset of cats, we cannot exclude the possibility that cats not examined in our study may display NAbs. However, it is remarkable that even cats harboring high titers of AAV-binding antibodies do not display neutralizing ability. Instead, the antibody-harboring cat sera has the potential to enhance infection in an AAV serotype-dependent manner.

On the surface, it might be difficult to reconcile the results of the two studies. One potential explanation resides in the fact that the NAb assay systems used in the two separate studies were different. Although the principle of NAb assays is the same across laboratories, there remains no standardized method to determine the existence of AAV NAbs. For example, NAb titers, which are normally expressed as the extent of serum or plasma sample dilutions that can suppress >50% transduction in reporter cells, can vary depending on how many AAV particles are used in the NAb assay system. Li *et al*.^20^ used a lower multiplicity of infection (MOI) in their NAb assay compared to ours, which might have resulted in a higher sensitivity than in our assay. They used an MOI of 2 × 10^3^ for AAV2 and AAV6 and an MOI of 1 × 10^4^ for AAV9 while we used an MOI of approximately 2.5 × 10^4^ for all the serotypes in our assay. For this reason, we additionally performed our NAb assay using the matched MOI, 2 × 10^3^, for AAV2 and AAV6. The result showed that there were no AAV2 or AAV6 NAbs in our AAV-binding antibody positive cat serum samples (Supplementary Fig. S2). The transduction-enhancing effects on AAV6 and the lack of such effect on AAV2 were also reproduced when using the matched MOI. Based on these considerations, we suspect that the difference between our study and that of Li *et al*. is not due to the assays employed to detect neutralizing activity. Instead, it might be attributed to the cat populations assessed in these two studies as they reside in two distant, unconnected continents. Supporting this idea is the unexpected finding reported by Li *et al*.^20^ that significant geographic differences in the prevalence of NAb can exist even within a country as small as Switzerland. An alternative, but not exclusive, possibility is that the different cat breeds used in the two studies are responsible for the divergent results. Further studies are necessary to distinguish between these two possibilities.

High prevalence of anti-AAV humoral immunity against many AAV serotypes in client-owned cats, feral cats and even SPF cats raises an interesting question as to how cats acquire such immunity against AAV. Although we do not have a clear explanation, several possibilities can be entertained. One is that cats are frequently exposed to AAV from human populations because cats are one of the most common companion animals. Humans worldwide are commonly infected with various AAV serotypes^25^. It is noteworthy that client-owned cats that should have more intensive interactions with humans than feral and SPF cats had AAV-binding antibodies that react more broadly with multiple serotypes than those found in feral and SPF cats. Another possibility is that antibodies raised against one or a few parvoviruses cross-reacted with other parvoviruses or AAV serotypes as exemplified by the anti-FPV antibodies that cross-react with AAV6 in our study. It has been well established that anti-AAV antibodies developed in humans show varying degrees of cross-reactivity across different AAV serotypes^28–30^. Thus, it remains possible that the high prevalence of anti-AAV antibodies found in cats is unrelated to the high prevalence of natural infection with multiple AAV serotypes. Further studies are needed to understand the potential mechanisms underlying these and other possibilities.

Non-neutralizing anti-AAV antibodies could develop upon AAV infection but such antibodies normally do not dominate, and the levels of immunoglobulins specific to AAV and titers of anti-AAV NAbs generally correlate well^31^. Fitzpatrick *et al*. has recently reported that only a small fraction of humans seropositive for AAV8 carry non-neutralizing AAV8-binding antibodies that dominate over NAbs (if indeed present) in the blood^23^. Although these authors could not define the origin of the non-neutralizing antibodies, they have suggested that the distinct biological nature of such non-neutralizing antibodies in part might stem from cross-reactivity of antibodies raised against closely-related, but different, viruses. The exceptionally high prevalence of non-neutralizing antibodies binding to multiple AAV serotypes in cats in our study supports this notion because, if cats had indeed encountered multiple AAV serotypes, they would have very likely developed NAbs against the encountered serotypes that should dominate over non-neutralizing antibodies.

Certain components present in animal and human sera have been identified as inhibitors or enhancers acting on AAV vector transduction in an animal species-dependent, AAV serotype-dependent, and target cell-dependent manner. Denard *et al*. have shown that galectin 3 binding protein (G3PB) in human and dog sera, but not mouse or monkey G3PB, inhibits AAV6 vector transduction^32^. They have also shown that C-reactive protein (CRP) in mouse sera but not that in human sera enhances AAV1 and AAV6 vector transduction^32^ while mouse CRP has no effect on AAV8 or AAV9 vector transduction^33^. A more recent study from the same group has identified mouse platelet factor 4 (PF4) as an inhibitor to AAV8 and AAV9 vector-mediated cardiac transduction in mice while it has no effect on transduction in the liver and skeletal muscles^34^. Human serum albumin has also been identified as an AAV transduction enhancer^35,36^. It appears that serum components interfering with AAV transduction are prevalent in a variety of animal species although the precise identity of these components is yet to be determined^37^. In this context, our study has indicated for the first time that cats also harbor a serum component that can enhance AAV6 vector transduction to a modest degree. Intriguingly, our results show that development and increase of serum enhancers in cats for AAV6 and AAV9 vector transduction coincide with elevated levels of AAV6 and AAV9-binding antibodies. Thus, it is not unreasonable to presume that non-neutralizing AAV6 and AAV9-binding immunoglobulins, in particular IgG that we measured in our study, have an AAV transduction-enhancing effect. Notably, the non-neutralizing AAV8-binding IgG antibodies found in a small fraction of humans also exhibit similar enhancing effects^23^. Nonetheless, alternative explanations are possible, including induction or depletion of non-IgG enhancers or inhibitory factors, respectively, associated with anti-viral immunity in cats. Future experiments using cat sera depleted of immunoglobulins will help understand the degree of IgG contributions to the AAV vector transduction-enhancing effects of the naïve and anti-AAV binding antibody-containing cat sera.

Cross-reactivity of anti-FPV antibodies with AAV6 capsids is unambiguous due to the observed coincidental emergence and elevations of anti-FPV NAbs and AAV6-binding antibodies in an SPF cat cohort that received the first and second FPV vaccination on the same dates. AAV6 and AAV1 capsids have a 99% amino acid sequence identity and are structurally very similar. Anti-AAV1 capsid mouse moAbs (ADK1a, ADK1b, 4E4 and 5H7) cross-react with AAV6 capsids^38,39^. These mouse moAb epitopes on the AAV capsids are: 448, 450, 453–457, and 500 for ADK1a; 256, 258–259, 261, 263–266, 272, 385–386, 547, 709–710, 716–718, 720, and 722 for ADK1b; 456–459 and 492–498 for 4E4; and 494, 496–499, 582–583, 588–595, 597 are for 5H7 (the numbers indicate the amino acid positions in AAV1 and AAV6 VP1 proteins)^38,39^. In this study, we deduced from the structural and bioinformatic information that the feline AAV6-binding antibodies target the epitope containing K531 and that L584 and V598 are not a part of the epitope despite the fact that all these three amino acids reside on the surface of the capsid. It should be noted that 531 is not a part of the epitopes recognized by moAbs that bind both AAV1 and AAV6, and 584 and 598 are directly adjacent to these epitopes. Anti-AAV6 capsid mouse moAbs (ADK6), which does not cross-react with the AAV1 capsid, recognizes an AAV6 capsid epitope containing K531^40^. Bennett *et al*. has shown that ADK6 binds AAV6 and AAV1E531K (an AAV1 mutant carrying an AAV6 amino acid at 531) while AAV1 and AAV6K531E escape ADK6 recognition. A similar profile of protein interactions with AAV1, AAV6 and their swapping mutants (AAV1E531K and AAV6K531E) has also been demonstrated with human G3BP^32^. These lines of evidence strongly indicate that the amino acid position 531 in AAV1 and AAV6 capsids is the critical interface of these differential virus-host protein interactions of the two closely-related AAV serotypes. Taken together, although experimental validation will be necessary to reach a definitive conclusion, it is most likely that the K531-containing capsid region is the epitope on the AAV6 capsid recognized by anti-FPV antibodies.

In summary, we report here high prevalence of anti-AAV humoral immunity in cats living in the Northeastern United States and show that they are not necessarily inhibitory to at least AAV2, 6 and 9. It was previously reported that FPV vaccination could induce antibodies against canine parvovirus (CPV)^41^ and our study adds to this knowledge by demonstrating that the antibodies raised by FPV vaccine also cross-react with AAV6. Considering that AAV9 is a robust serotype for *in vivo* transduction in various animal species including cats and that pre-existing anti-AAV9 humoral immunity, even if present, enhances AAV9 transduction, AAV9 or its-derived mutants will offer a compelling gene delivery vehicle for feline gene therapy and preclinical evaluation using feline models^10,42–45^.

## Methods

### Cells

Chinese hamster ovary (CHO) K1 cells (CHO-K1, RRID:CVCL_0214) were purchased from ATCC. CHO-Lec2 (Lec2) cells were kindly gifted by Dr. Aravind Asokan (Duke University). CHO-K1 and CHO-Lec2 cells were grown in F-12K medium (12–615 F, Lonza, Basel, Switzerland) and Alpha-Minimum Essential Medium (Alpha-MEM: M8042-500ML, MilliporeSigma, St. Louis, MO), respectively. The media were supplemented with 10% fetal bovine serum (FBS), L-glutamine and penicillin–streptomycin. Frozen cell stocks were created without further authentication from the original vials we received before use for experiments^46^.

### AAV vectors

AAV1 through AAV11 vectors were produced in human embryonic kidney HEK 293 cells (AAV-293, Agilent, RRID: CVCL_6871) by an adenovirus-free plasmid transfection method and purified by two rounds of cesium chloride (CsCl) density-gradient ultracentrifugation followed by dialysis as described elsewhere^47^.

### Cat serum samples

No cats were used at Oregon Health & Science University. Cat serum samples were collected by the identified commercial vectors or from archival samples from the identified diagnostic lab. Sera of 35 client-owned cats were anonymous archival leftover samples that had been submitted by the clients’ veterinarians for clinical diagnostic assays, and were kindly provided by Dr. Edward Dubovi at the Animal Health Diagnostic Center, College of Veterinary Medicine, Cornell University in New York. Sera of 20 feral cats living in Lancaster County in Pennsylvania State were purchased from BioChemed Services, Winchester, VA. BioChemed Services Inc. sells blood serum from various animals. Their blood collection methods are approved by their Quality Assurance Team, BioChemed Services Inc. and the facility is USDA inspected. Age and gender information was not available for the client-owned and feral cat groups. Sera of a total 44 SPF cats were purchased from Liberty Research Inc, Waverly, NY. They were all domestic shorthair mixed breed cats: 15 males at the age of 4 to 10 months and 15 females aged 4 to 8 months and all received Fel-O-Vax (Boehringer Ingelheim, Ridgefield, CT) once to three times (once for 1 cat, twice for 23 cats, and three times for 6 cats); and a cohort of 7 males and 7 females that were born on dates that differ from each other by 5 days or less, and were at 5 months of age. Sera of this cohort were collected one day before Fel-O-Vax vaccine administration (Day 0), and at Day 20 and Day 36. At Day 22, the cohort received Fel-O-Vax again as a booster. All animal procedures including collection of the sera purchased from Liberty Research Inc. were conducted in accordance with the relevant guidelines and regulations and approved by the Institutional Animal Care and Use Committee, Liberty Research Inc. (now Marshall Bioresources Inc.). Liberty Research Inc. was fully accredited by AAALAC at the time of the sample collection.

### AAV-binding antibody enzyme-linked immunosorbent assay (ELISA)

CsCl-purified AAVx-CMV-lacZ or AAVx-EF1α-nlslacZ vectors (x = serotypes 1 to 11)^48^ were diluted in Coating Buffer (100 mM sodium carbonate-bicarbonate buffer) to 2 × 10^10^ vector genome (vg)/mL. Nunc MaxiSorp flat-bottom 96-well plates (439454, Thermo Fisher Scientific, Waltham, MA) were coated with 50 μl of the diluted AAV vector solution, sealed with an adhesive film and incubated at 37 °C for 1 h or at 4 °C overnight. The plates were then washed three times with 300 μL of Washing Buffer (0.05% Tween 20 in phosphate-buffered saline (PBS)) and blocked with 200 μL of Sample Buffer (5% bovine serum albumin in PBS) at 37 °C for 1 h. After the plates were washed once with Washing Buffer, 50 μL of each sample containing cat serum diluted at 1:5 was added to each well and incubated at 37 °C for 1 h. After the plates were washed five times with Washing Buffer, 100 μL Sample Buffer containing 1:5,000-diluted horseradish peroxidase (HRP)-conjugated goat anti-cat IgG antibody (sc-2900, Santa Cruz Biotechnology, Dallas, TX, RRID: AB_650489) was added to each well and incubated at 37 °C for 1 h. The plates were washed again five times, and 100 μL of 0.1 M sodium citrate solution containing o-phenylenediamine dihydrochloride substrate (P4664-100TAB, MilliporeSigma) and 0.0064% hydrogen peroxide was added to each well. After incubation at room temperature for 8 min, 50 μL of Stop Solution (2.5 M sulfuric acid) was added to each well, and the absorbance was determined at 490 nm and expressed as optical density (OD) values.

Due to potential plate-to-plate fluctuations of OD values, we employed corrected OD (ODc) values so that we could compare OD values across different 96-well plates. For this purpose, we created a reference cat serum using the cat sera we obtained. Each plate had 4 wells that were coated with 50 μL of 1:100-diluted reference cat serum in Coating Buffer. These 4 wells, which were coated with the reference cat serum but not with AAV, underwent the same ELISA procedures with Sample Buffer only rather than diluted cat serum-containing Sample Buffer. An average of the OD values from the 4 reference wells in a plate was determined for each plate and used to cancel out small differences in OD values between the plates.

The threshold ODc values that defined seropositivity for each serotype were determined in the following manner to binarize the dataset. As described in the Results section, k-means non-hierarchical clustering and t-SNE independently identified a distinct cluster comprising exclusively Group 4 Pre-vac cats. Because of this distinct nature of Group 4 Pre-vac and the fact that majority of the Group 4 Pre-vac cats showed ODc values among the lowest, we reasoned that the majority of that Group 4 Pre-vac cats had not yet mounted humoral immunity against many AAV serotypes and therefore the ODc values in Group 4 Pre-vac represent those of bona-fide negative controls when outliers are removed. With this rationale, we first excluded outliers showing values more than three times the interquartile range (IQR) beyond the upper quartile (Q3), which resulted in exclusion of 7 from the 154 ODc values in this group (*i.e*., 11 serotypes and 14 cats). Using the dataset of the 147 ODc values that were not excluded, we determined the cut-off value as Q3 + 1.5IQR for each AAV serotype. We defined seropositive cats as those that have ODc values more than the cut-off value for each AAV serotype. The absence of inhibitory antibodies in cats defined as the bona-fide negative controls was confirmed for AAV2, AAV6 and AAV9 by the anti-AAV NAb assay using 4 Group 4 Pre-vac cat samples, respectively.

### Anti-FPV neutralizing antibody (NAb) titration

Anti-FPV NAb titers were determined by a Feline Parvovirus (Panleukopenia) Virus Hemagglutination Inhibition assay offered as a commercial service by Cornell University, Animal Health Diagnostic Center. For more information of this assay, refer to https://www.vet.cornell.edu/animal-health-diagnostic-center/laboratories.

### *In vitro* anti-AAV neutralizing antibody (NAb) assay

We carried out NAb assays as previously described with minor modifications^25^. In brief, we used CHO-K1 cells for AAV2 and AAV6 and CHO-Lec2 cells for AAV9 as reporter cells. Reporter cells were seeded at a density of 4 × 10^4^ cells per well on white-walled, clear-bottomed, 96-well plates (3610; Corning Incorporated, Kennebunk, ME) on 1 day before the assay. Serum samples were heat-inactivated at 56 °C for 1 h and diluted in serum-free corresponding media at 2.5, 5 and 40-fold dilutions. CsCl-purified AAVx-CMV-luciferase (luc) vector (x = serotypes 2, 6 or 9)^47^ was diluted in serum-free media to 1 × 10^11^ vg/mL. The diluted serum samples were mixed with AAV vector solution at a 1:1 volume ratio (initial serum dilution, 1:2.5) and incubated for 1 h at 37 °C. The reporter cells were pre-treated with the wild-type human adenovirus type 5 (Ad5) at an MOI of 10 for 1 h in a serum-free condition. Twenty μL of each serum-vector mixture containing 1 × 10^9^ vg of the vector was added to each well of 96-well plates, and incubated for 2 days. The approximate MOI under these conditions is 2.5 × 10^4^. Luciferase expression levels were quantified with the Bright-Glo Luciferase Assay System (E2610, Promega, Madison, WI) using the Synergy 2 microplate reader (BioTek, Winooski, VT) according to the manufacture’s protocol. Relative transduction efficiencies of the reporter cells with AAVx-CMV-luc vector that had been pre-incubated with diluted sera versus with no-serum control were determined based on a standard curve generated with the following four standards: 25%, 50%, 100% and 200% of the no-sera control. NAb titer was defined as the highest dilution that suppressed the relative transduction by >50%. For a limited number of samples, we additionally used an 8 × 10^7^ of AAV vector-per-well condition that matches with the MOI used in Li *et al*.’s study^20^. We have confirmed that our anti-AAV NAb assay could effectively detect feline anti-AAV2, AAV6 and AAV9 NAb in sera collected from two cats that were injected intravenously with an AAV library containing various AAV serotypes including AAV2, AAV6 and AAV9. These samples were obtained in a separate study that was approved by the Oregon Health & Science University (OHSU) Institutional Animal Care and Use Committee (IACUC).

### Clustering and dimensional reduction of data

To identify groups of cats that share similar spectrum of AAV-binding antibodies against multiple serotypes in an unbiased fashion, we employed k-means non-hierarchical clustering and t-distributed stochastic neighbor embedding (t-SNE). To determine an optimal number of k-means clustering, we computed the total within-cluster sum of square (WSS) values with k = 1 to 15. We plotted the WSS and k values on a graph and identified an elbow point in the plot as an optimal k. We used the following 4 algorithms: Lloyd’s, Forgy’s, MacQueen’s, and Hartigan-Wong’s. We restarted each algorithm 100 times with random initialization to increase the accuracy, which yielded exactly the same clustering results with all the four algorithms. To visualize each cat’s profile of anti-AAV humoral immunity in a two-dimensional space, t-SNE was employed using a range of perplexity values of 5 to 20, showing a consistent pattern comprising 5 clusters. The t-SNE plot shown in this paper used a perplexity value of 6.

### Molecular modeling of AAV and FPV capsid structures

Three-dimensional (3D) atomic structure data of AAV6 and FPV capsids were obtained from the Research Collaboratory for Structural Bioinformatics Protein Data Bank (RRID:SCR_012820) (PDB ID 4V86 and 1C8F, respectively). Using the Least-Squares (LSQ) Superposition function in COOT^49^ (Coot, RRID: SCR_014222), AAV6 and FPV capsid proteins were superposed and a pairwise alignment of capsid amino acid sequences was obtained based on 3D structures. Superposed structures were visualized by PyMOL (PyMOL, RRID: SCR_000305). We assessed similarity in topology between a local AAV6 capsid structure and the topologically corresponding FPV capsid structure by both visual inspection and measuring root-mean-square deviations (RMSDs) in Cα positions between the two local structures. Local structures we compared are: (1) AAV6 capsid structure composed of all the 17 outer surface-exposed amino acid residues within 12.5 Å in distance from the Cα of K531 and the topologically corresponding FPV capsid structure; and (2) AAV6 capsid structure composed of all the 22 outer surface-exposed amino acid residues within 12.5 Å in distance from the Cα of L584 and the topologically corresponding FPV capsid structure.

### Statistical analyses

For statistical analyses of continuous data, we used non-parametric tests because Shapiro–Wilk test for normality rejected the null hypothesis of normally with P < 0.05 in the majority of the datasets. We assessed statistical significance of observed differences between groups with a two-sided Mann-Whitney U-test or a two-sided Wilcoxon signed-ranked test. We used a two-sided Boschloo’s exact unconditional test to assess the degree of seropositivity between groups. We used Bonferroni correction for comparisons of multiple groups. We assessed correlation between two connected variables by a Spearman’s rank correlation test. Standard error of the mean (SEM) is used to show the dispersion of a dataset throughout the manuscript. We used R (CRAN, RRID: SCR_003005) for statistical analyses.

## Supplementary information

Supplementary Information.
