# Supplementary material for: Adeno-associated virus-binding antibodies detected in cats living in the Northeastern United States lack neutralizing activity
Source: Sci Rep. 2020 Jun 22;10:10073. doi: 10.1038/s41598-020-66596-4 (PMC7308316; doi:10.1038/s41598-020-66596-4)
Supplement: Supplementary file 1 — Supplementary Information. [file 41598_2020_66596_MOESM1_ESM.pdf]

SUPPLEMENTARY INFORMATION

Adeno-associated virus-binding antibodies detected in cats living in the Northeastern United States lack neutralizing activity

Kei Adachi[1], Gregory A. Dissen[2,4], Alejandro Lomniczi[2], Qing Xie[1], Sergio R. Ojeda[2], and Hiroyuki Nakai[1,2,3*]

Departments of [1]Molecular & Medical Genetics and [3]Molecular Microbiology & Immunology, Oregon Health & Science University School of Medicine, Portland, Oregon 97239; [2]Division of Neuroscience and [4]Molecular Virology Core, Oregon National Primate Research Center, Beaverton, Oregon 97006, United States of America.

Supplementary Figure S1. ODc values of AAV-binding antibody (Ab)-positive samples selected for anti-AAV NAb assays. (a) AAV2-binding Ab ELISA ODc values obtained from a total of 99 cats are sorted in a descending order from the left to the right. The AAV2-binding antibody-positive samples used for the anti-AAV2 neutralizing antibody (NAb) assay are indicated as solid black bars with the following animal IDs from the left to the right: G1-1, G1-18, G1-17, G1-8, G1-14, G2-16, G2-9, G2-2, G2-18, G2-12, G3-16, G3-26, G3-8, and G3-1. **(b)** The same graphic representation for AAV6-binding antibody ELISA ODc values. The AAV6-binding antibody-positive samples used for the anti-AAV6 NAb assay are indicated as solid black bars with the following animal IDs from the left to the right: G1-1, G1-8, G3-22, G3-18, G1-9, G3-14, G3-1, G1-3, G2-2, G4v-1, G4v2, G2-9, G4v-12, G4v-4, G2-13, and G2-16. **(c)** The same graphic representation for AAV9-binding antibody ELISA ODc values. The AAV9-binding antibody-positive samples used for the anti-AAV9 NAb assay are indicated as solid black bars with the following animal IDs from the left to the right: G1-31, G2-5, G1-25, G1-32, G1-33, G1-35, G1-12, G2-4, G3-8, G2-2, G2-1, G3-17, G3-12, and G3-15.

Supplementary Figure S2. Anti-AAV2 and AAV6-NAb assays at a lower MOI. (a) In vitro anti-AAV2 NAb assay was performed as described in Methods using an MOI of 2 x 10^3 to investigate the presence or absence of anti-AAV2 NAbs in cat sera. The Y-axis shows transduction efficiency of reporter cells with AAV2 vector pre-incubated with sample sera diluted at 1:2.5. Results of individual animals are shown with animal identification numbers (IDs). Values are averages of technical duplicates. ID Gx-y indicates x=Group number and y=animal number specific to each animal in each group. G4 and G4v indicates Group 4 Pre-vac and Post-vac animals, respectively. **(b)** The same assay was performed for anti-AAV6 NAbs.

Suppl Fig. S1

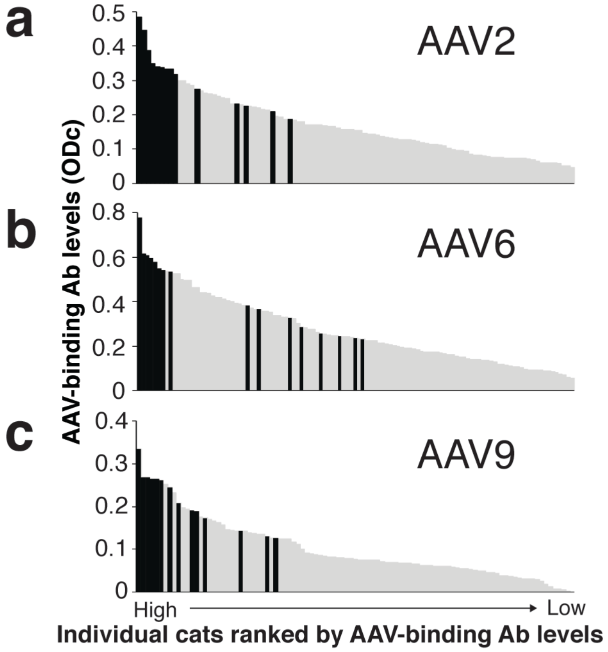
a
AAV2
0.5
0.4
0.3
0.2
0.1
0
b
AAV6
0.8
0.6
0.4
0.2
0
c
AAV9
0.4
0.3
0.2
0.1
0
AAV-binding Ab levels (ODc)
High
Low
Individual cats ranked by AAV-binding Ab levels

Suppl Fig. S2

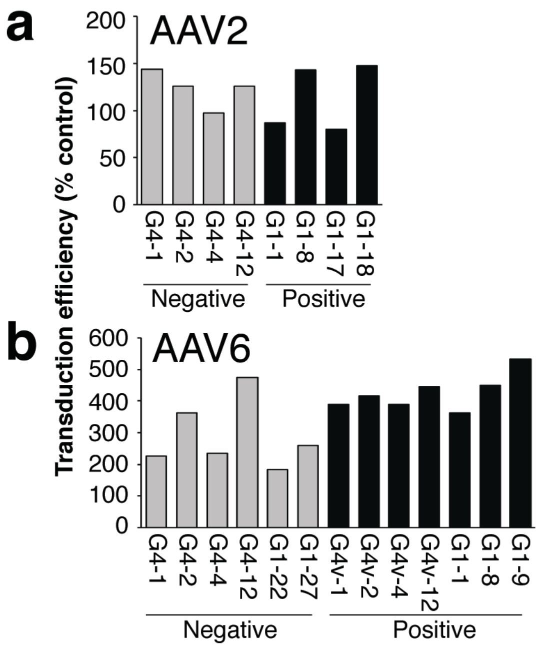
a
AAV2
200
150
100
50
0
G4-1
G4-2
G4-4
G4-12
G1-1
G1-8
G1-17
G1-18
Negative
Positive
b
AAV6
600
500
400
300
200
100
0
G4-1
G4-2
G4-4
G4-12
G1-22
G1-27
G4v-1
G4v-2
G4v-4
G4v-12
G1-1
G1-8
G1-9
Negative
Positive
Transduction efficiency (% control)

Cat ID	Gender	Age (months)	Breed	Source	No. of times of FPV Vaccinations	Days from the last Vaccination	Cluster ID (k-means)	Cluster ID (t-SNE)
G1-1				Cornell Univ., NY			Ek	Et
G1-2				Cornell Univ., NY			Ck	Ct
G1-3				Cornell Univ., NY			Dk	Et
G1-4				Cornell Univ., NY			Ck	Ct
G1-5				Cornell Univ., NY			Dk	Dt
G1-6				Cornell Univ., NY			Dk	Dt
G1-7				Cornell Univ., NY			Dk	Ct
G1-8				Cornell Univ., NY			Ek	Et
G1-9				Cornell Univ., NY			Ek	Et
G1-10				Cornell Univ., NY			Dk	Et
G1-11				Cornell Univ., NY			Dk	Dt
G1-12				Cornell Univ., NY			Dk	Et
G1-13				Cornell Univ., NY			Dk	Et
G1-14				Cornell Univ., NY			Dk	Dt
G1-15				Cornell Univ., NY			Dk	Et
G1-16				Cornell Univ., NY			Dk	Et
G1-17				Cornell Univ., NY			Ek	Et
G1-18				Cornell Univ., NY			Ek	Et
G1-19				Cornell Univ., NY			Ck	Ct
G1-20				Cornell Univ., NY			Dk	Dt
G1-21				Cornell Univ., NY			Ck	Ct
G1-22				Cornell Univ., NY			Ak	At
G1-23				Cornell Univ., NY			Ck	Ct
G1-24				Cornell Univ., NY			Dk	Et
G1-25				Cornell Univ., NY			Ck	Ct
G1-26				Cornell Univ., NY			Ak	At
G1-27				Cornell Univ., NY			Ak	At
G1-28				Cornell Univ., NY			Ak	At
G1-29				Cornell Univ., NY			Ak	At
G1-30				Cornell Univ., NY			Ck	Ct
G1-31				Cornell Univ., NY			Ck	Ct
G1-32				Cornell Univ., NY			Dk	Et
G1-33				Cornell Univ., NY			Dk	Ct
G1-34				Cornell Univ., NY			Ck	Ct
G1-35				Cornell Univ., NY			Dk	Et
G2-1				Biochem Serv., PA			Ak	At
G2-2				Biochem Serv., PA			Dk	Et
G2-3				Biochem Serv., PA			Ck	Ct
G2-4				Biochem Serv., PA			Ck	Ct
G2-5				Biochem Serv., PA			Ck	Ct
G2-6				Biochem Serv., PA			Ck	Ct
G2-7				Biochem Serv., PA			Ak	Ct
G2-8				Biochem Serv., PA			Ck	Ct
G2-9				Biochem Serv., PA			Dk	Dt
G2-10				Biochem Serv., PA			Ck	Ct
G2-11				Biochem Serv., PA			Ck	Ct
G2-12				Biochem Serv., PA			Dk	Dt
G2-13				Biochem Serv., PA			Ck	Ct
G2-14				Biochem Serv., PA			Ck	Ct
G2-15				Biochem Serv., PA			Ck	Ct
G2-16				Biochem Serv., PA			Dk	Dt
G2-17				Biochem Serv., PA			Ck	Ct
G2-18				Biochem Serv., PA			Dk	Dt
G2-19				Biochem Serv., PA			Ck	Ct
G2-20				Biochem Serv., PA			Ck	Ct
G3-1	F	4.3	DSH	Liberty Res., NY	2	8	Bk	Bt
G3-2	F	5.8	DSH	Liberty Res., NY	2	8	Bk	Bt
G3-3	F	5.2	DSH	Liberty Res., NY	1	34	Bk	Bt
G3-4	F	4.3	DSH	Liberty Res., NY	2	8	Ck	Bt
G3-5	F	5.1	DSH	Liberty Res., NY	2	8	Bk	Dt
G3-6	F	6.5	DSH	Liberty Res., NY	2	8	Bk	Bt
G3-7	F	4.5	DSH	Liberty Res., NY	2	8	Bk	Bt

G3-8	F	4.3	DSH	Liberty Res., NY	2	8	Dk	Bt
G3-9	F	5.9	DSH	Liberty Res., NY	2	8	Bk	Bt
G3-10	F	6.0	DSH	Liberty Res., NY	2	8	Bk	Bt
G3-11	F	7.1	DSH	Liberty Res., NY	2	8	Bk	Bt
G3-12	F	4.4	DSH	Liberty Res., NY	2	8	Bk	Bt
G3-13	F	5.7	DSH	Liberty Res., NY	2	8	Bk	Bt
G3-14	F	5.1	DSH	Liberty Res., NY	2	8	Bk	Bt
G3-15	F	7.5	DSH	Liberty Res., NY	2	8	Dk	Dt
G3-16	M	8.4	DSH	Liberty Res., NY	2	152	Bk	Dt
G3-17	M	8.7	DSH	Liberty Res., NY	2	152	Bk	Bt
G3-18	M	8.2	DSH	Liberty Res., NY	3	99	Bk	Bt
G3-19	M	3.6	DSH	Liberty Res., NY	1	33	Ak	At
G3-20	M	9.6	DSH	Liberty Res., NY	2	153	Bk	Bt
G3-21	M	8.0	DSH	Liberty Res., NY	3	99	Ak	At
G3-22	M	7.9	DSH	Liberty Res., NY	3	95	Bk	Bt
G3-23	M	8.4	DSH	Liberty Res., NY	2	144	Ak	At
G3-24	M	8.3	DSH	Liberty Res., NY	2	144	Ak	At
G3-25	M	8.7	DSH	Liberty Res., NY	2	153	Bk	Bt
G3-26	M	8.0	DSH	Liberty Res., NY	3	99	Bk	Ct
G3-27	M	3.6	DSH	Liberty Res., NY	1	33	Ak	At
G3-28	M	8.4	DSH	Liberty Res., NY	2	154	Ak	At
G3-29	M	4.5	DSH	Liberty Res., NY	2	68	Ak	At
G3-30	M	8.3	DSH	Liberty Res., NY	3	99	Bk	Bt
G4-1	F	4.9	DSH	Liberty Res., NY	2	14	Ak	At
G4-2	F	4.9	DSH	Liberty Res., NY	2	14	Ak	At
G4-3	M	4.9	DSH	Liberty Res., NY	2	14	Ak	At
G4-4	M	4.9	DSH	Liberty Res., NY	2	14	Ak	At
G4-5	F	4.8	DSH	Liberty Res., NY	2	14	Ak	At
G4-6	F	4.8	DSH	Liberty Res., NY	2	14	Ak	At
G4-7	M	4.8	DSH	Liberty Res., NY	2	14	Ak	Ct
G4-8	F	4.8	DSH	Liberty Res., NY	2	14	Ck	Ct
G4-9	F	4.8	DSH	Liberty Res., NY	2	14	Ak	At
G4-10	M	4.8	DSH	Liberty Res., NY	2	14	Ak	At
G4-11	M	4.8	DSH	Liberty Res., NY	2	14	Ak	At
G4-12	M	4.7	DSH	Liberty Res., NY	2	14	Ak	At
G4-13	F	4.7	DSH	Liberty Res., NY	2	14	Ak	At
G4-14	M	4.7	DSH	Liberty Res., NY	2	14	Ek	At

Supplementary Table S1. Information of the cats used in this study. Cat IDs are composed of the group names followed by each cat-specific identification numbers (IDs) in each group, linked with a hyphen between the group names and cat-specific IDs. G1, Group 1; G2, Group 2; G3, Group 3 and G4, Group 4. DSH, domestic shorthair mixed breed cats. For Cluster IDs, please refer to the main text. Blank cells indicate that no information was available. NY, New York State; PA, Pennsylvania State.

Cluster ID (t-SNE)	Cluster ID (k-means clustering)					
	**Ak**	**Bk**	**Ck**	**Dk**	**Ek**	**Total**
**At**	24	0	0	1	0	25
**Bt**	1	17	1	1	0	20
**Ct**	2	2	22	1	0	27
**Dt**	0	1	1	9	0	11
**Et**	0	0	0	11	5	16
**Total**	27	20	24	23	5	99

Supplementary Table S2. A comparison between clusters of the 99 cat serum samples identified by k-means clustering and those visualized in a two-dimensional space by t-SNE. For the definition of Cluster IDs (i.e., At, Ak, etc.), please refer to the main text.

No. of Ab-reacting AAV serotypes	Client-owned cats with AAV-binding Abs (n=32)		Feral cats with AAV-binding Abs (n=20)		P value (* statistically significant)
	No. of cats	Percentage	No. of cats	Percentage	
**1**	3	9	1	5	1
**2 or less**	3	9	5	25	0.5028
**3 or less**	4	13	9	45	0.03621*
**4 or less**	7	22	11	55	0.05181
**5 or less**	10	31	12	60	0.14352
**6 or less**	10	31	14	70	0.022314*
**7 or less**	11	34	15	75	0.016134*
**8 or less**	12	38	15	75	0.026613*
**9 or less**	14	44	15	75	0.08784
**10 or less**	25	78	17	85	1
**11 or less**	32	100	20	100	1
**No. of Ab-reacting AAV serotypes**	**Client-owned cats with AAV-binding Abs (n=32)**		**Feral cats with AAV-binding Abs (n=20)**		**P value (* statistically significant)**
	**No. of cats**	**Percentage**	**No. of cats**	**Percentage**	
**1 or more**	32	100	20	100	1
**2 or more**	29	91	19	95	1
**3 or more**	29	91	15	75	0.5028
**4 or more**	28	88	11	55	0.03621*
**5 or more**	25	78	9	45	0.05181
**6 or more**	22	69	8	40	0.14352
**7 or more**	22	69	6	30	0.022314*
**8 or more**	21	66	5	25	0.016134*
**9 or more**	20	63	5	25	0.026613*
**10 or more**	18	56	5	25	0.08784
**11**	7	22	3	15	1

Supplementary Table S3. The degree of broad reactivity of AAV-binding antibodies (Abs) in client-owned and feral cat populations. The extent of broad reactivity of AAV-binding antibodies was assessed by the number of antibody-reacting AAV serotypes in each cat carrying AAV binding antibodies. A statistical comparison of the degree of broad reactivity between client-owned cats and feral cats was done using a two-sided Boschloo's exact unconditional test. Please note that the top and bottom comparisons are reciprocal to each other, providing exactly same P values for each corresponding comparison. Bonferroni-corrected P values are shown.

No. of Ab-reacting AAV serotypes	Client-owned cats with AAV-binding Abs (n=32)		SPF cats with AAV-binding Abs (n=27)		P value (* statistically significant)
	No. of cats	Percentage	No. of cats	Percentage	
**1**	3	9	4	15	1
**2 or less**	3	9	9	33	0.08274
**3 or less**	4	13	14	52	0.00339*
**4 or less**	7	22	15	56	0.03279*
**5 or less**	10	31	19	70	0.008607*
**6 or less**	10	31	22	81	0.0002874*
**7 or less**	11	34	23	85	0.0002481*
**8 or less**	12	38	25	93	0.00001971*
**9 or less**	14	44	26	96	0.00002265*
**10 or less**	25	78	26	96	0.16884
**11 or less**	32	100	27	100	1

No. of Ab-reacting AAV serotypes	Client-owned cats with AAV-binding Abs (n=32)		SPF cats with AAV-binding Abs (n=27)		P value (* statistically significant)
	No. of cats	Percentage	No. of cats	Percentage	
**1 or more**	32	100	27	100	1
**2 or more**	29	91	23	85	1
**3 or more**	29	91	18	67	0.08274
**4 or more**	28	88	13	48	0.00339*
**5 or more**	25	78	12	44	0.03279*
**6 or more**	22	69	8	30	0.008607*
**7 or more**	22	69	5	19	0.0002874*
**8 or more**	21	66	4	15	0.0002481*
**9 or more**	20	63	2	7	0.00001971*
**10 or more**	18	56	1	4	0.00002265*
**11**	7	22	1	4	0.16884

Supplementary Table S4. The degree of broad reactivity of AAV-binding antibodies (Abs) in client-owned and SPF cat populations. The extent of broad reactivity of AAV-binding antibodies was assessed by the number of antibody-reacting AAV serotypes in each cat carrying AAV binding antibodies. A statistical comparison of the degree of broad reactivity between client-owned cats and SPF cats was done using a two-sided Boschloo's exact unconditional test. Please note that the top and bottom comparisons are reciprocal to each other, providing exactly same P values for each corresponding comparison. Bonferroni-corrected P values are shown.

No. of Ab-reacting AAV serotypes	Feral cats with AAV-binding Abs (n=20)		SPF cats with AAV-binding Abs (n=27)		P value (* statistically significant)
	No. of cats	Percentage	No. of cats	Percentage	
**1**	1	5	4	15	1
**2 or less**	5	25	9	33	1
**3 or less**	9	45	14	52	1
**4 or less**	11	55	15	56	1
**5 or less**	12	60	19	70	1
**6 or less**	14	70	22	81	1
**7 or less**	15	75	23	85	1
**8 or less**	15	75	25	93	0.4086
**9 or less**	15	75	26	96	0.14511
**10 or less**	17	85	26	96	0.8247
**11 or less**	20	100	27	100	1
**No. of Ab-reacting AAV serotypes**	**Feral cats with AAV-binding Abs (n=20)**		**SPF cats with AAV-binding Abs (n=27)**		**P value (* statistically significant)**
	**No. of cats**	**Percentage**	**No. of cats**	**Percentage**	
**1 or more**	20	100	27	100	1
**2 or more**	19	95	23	85	1
**3 or more**	15	75	18	67	1
**4 or more**	11	55	13	48	1
**5 or more**	9	45	12	44	1
**6 or more**	8	40	8	30	1
**7 or more**	6	30	5	19	1
**8 or more**	5	25	4	15	1
**9 or more**	5	25	2	7	0.4086
**10 or more**	5	25	1	4	0.14511
**11**	3	15	1	4	0.8247

Supplementary Table S5. The degree of broad reactivity of AAV-binding antibodies (Abs) in feral and SPF cat populations. The extent of broad reactivity of AAV-binding antibodies was assessed by the number of antibody-reacting AAV serotypes in each cat carrying AAV binding antibodies. A statistical comparison of the degree of broad reactivity between feral cats and SPF cats was done using a two-sided Boschloo's exact unconditional test. Please note that the top and bottom comparisons are reciprocal to each other, providing exactly same P values for each corresponding comparison. Bonferroni-corrected P values are shown.

Groups of cats	No. of serotype-serotype combinations showing linkage values of seropositivity for each category	
	Linkage values ≤ 3 (low to moderate)	Linkage values > 3 (high)
**Group 1 (client-owned)**	49	61
**Group 2 (feral)**	63	47
**Group 3 (SPF)**	75	35

Supplementary Table S6. Overall degree of seroreactivity linkage of AAV-binding antibodies between different serotypes in each cat group. A total of 110 seroreactivity linkage values in each animal group (Fig. 2) are categorized into two groups, one with values of 3 and less and the other with values of 3 or more. The number of each category between two groups was statistically compared between two groups. P=0.020 for Group 1 vs. Group 2, P=0.30 for Group 2 vs. Group 3, and P=0.0012 for Group 1 vs. Group 3. P values were determined by a two-sided Boschloo's exact unconditional test followed by Bonferroni correction.

Serotypes	Bonferroni-corrected P values in each comparison					
	Comparison in the prevalence of antibodies (Boschloo)			Comparison in the levels of antibodies (Mann-Whitney)		
	G1 vs. G2	G1 vs. G3	G2 vs. G3	G1 vs. G2	G1 vs. G3	G2 vs. G3
**AAV1**	0.50	0.10	1	0.014*	1.8×10^{-3}**	0.77
**AAV2**	0.86	1.3×10^{-3}**	2.1×10^{-4}***	0.017*	1.1×10^{-8}*****	7.9×10^{-5}****
**AAV3**	0.03*	1.1×10^{-6}*****	0.15	0.049*	2.2×10^{-3}**	1
**AAV4**	0.25	4.0×10^{-3}**	0.82	0.06	0.14	8.8×10^{-5}****
**AAV5**	0.15	4.3×10^{-6}*****	0.045*	0.11	1.1×10^{-3}**	0.61
**AAV6**	0.24	1	0.044*	0.13	2.0×10^{-5}****	9.2×10^{-3}**
**AAV7**	0.15	1.6×10^{-3}**	0.82	0.19	1.3×10^{-7}*****	1.5×10^{-4}***
**AAV8**	1	0.26	0.12	0.33	0.029*	0.87
**AAV9**	1	0.014*	0.10	1	4.0×10^{-4}***	8.8×10^{-5}****
**AAV10**	0.59	1	0.34	1	0.37	0.36
**AAV11**	0.13	0.033*	1	1	0.15	4.8×10^{-3}**

Supplementary Table S7. Comparisons of the prevalence and levels of AAV-binding antibodies to each serotype between the groups. Multiple pair-wise comparisons were performed for each serotypes between the three groups. A two-sided Boschloo's exact unconditional test and a two-sided Mann-Whitney U-test were used as indicated. Statistically significant P values are indicated with asterisks (*P<0.05, **P<0.01; ***P<0.001, ****P<0.0001, *****P<0.00001). G1, Group 1 (client-owned cats); G2, Group 2 (feral cats); and G3, Group 3 (FPV-vaccinated SPF cats).

Serotype comparison	P values corrected for multiple comparisons (Bonferroni)	
	Antibody prevalence (n=30)	AAV-binding antibody levels (n=30)
AAV6 vs. AAV1	0.0041	9.0×10^{-6}
AAV6 vs. AAV2	0.0041	2.0×10^{-7}
AAV6 vs. AAV3	2.2×10^{-7}	2.0×10^{-7}
AAV6 vs. AAV4	4.2×10^{-5}	3.1×10^{-7}
AAV6 vs. AAV5	2.2×10^{-8}	1.0×10^{-7}
AAV6 vs. AAV7	4.2×10^{-5}	2.0×10^{-7}
AAV6 vs. AAV8	1	1.0×10^{-7}
AAV6 vs. AAV9	4.2×10^{-5}	1.0×10^{-7}
AAV6 vs. AAV10	0.75	1.1×10^{-5}
AAV6 vs. AAV11	0.011	2.0×10^{-7}

Supplementary Table S8. Statistical comparisons of the prevalence and the levels of AAV-binding antibodies between AAV6 and other serotypes in Group 3 cats. A two-sided Boschloo's exact unconditional test and a Wilcoxon signed-rank test were used to statistically compare the prevalence and the quantities of antibody levels, respectively, between two serotype pairs. P values were determined by 55 multiple comparisons and adjusted by the Bonferroni correction.

Aligned amino acid residues		Distance between Cα positions	Outer surface-exposed?
**AAV6 (Distance from K531*, Å)**	**FPV**		
K531 (0)	D367	2.22	Yes
D530 (3.77)	T366	6.22	Yes
D532 (3.84)	E368	5.19	Yes
D529 (4.81)	Q365	5.41	Yes
K528 (5.78)	A364	3.56	Yes
H527 (6.11)	G363	5.05	Yes
K533 (6.68)	N369	8.97	Yes
F534 (6.68)	Q370	4.63	Yes
S526 (9.35)	G362	5.49	Yes
F535 (9.8)	A371	6.04	Yes
G513 (9.89)**	Q350	6.14	Yes
S490 (9.9)			Yes
N512 (10.53)**	T349	1.85	Yes
R488 (11.2)			Yes
T574 (11.46)	P410	4.71	Yes
V489 (11.51)			No
K491 (11.51)			Yes
E575 (11.51)	E411	3.96	Yes
V572 (11.52)	R408	1.62	Yes
N383 (12.01)**	D215	1.86	Yes
E564 (12.05)	H403	3.35	Yes
A525 (12.07)	G360	3.41	No
RMSD (surface-exposed amino acids only) = 4.87 Å			

Supplementary Table S9. Differences in topology around the AAV6 K531 and FPV D367 regions. *Distances from AAV6 K531 in Cα positions. **They are from neighboring subunits.

Aligned amino acid residues		Distance between Cα positions	Outer surface-exposed?
AAV6 (Distance from L584*, Å)	FPV		
L584 (0)	I418	6.08	Yes
Q585 (3.81)	N419	5.37	No
N583 (3.87)	N417	6.67	Yes
R488 (5.46)**	Y342	9.30	Yes
S586 (5.76)	F420	12.54	Yes
Q487 (5.98) **	P341	9.41	No
D590 (6.45)	G441	4.01	Yes
S588 (7.12)	K439	11.05	Yes
P591 (7.28)	I442	5.10	Yes
V582 (7.49)	Q416	7.64	Yes
T574 (7.64) **	P410	4.71	Yes
V489 (7.97) **			No
E575 (8.27) **	E411	3.96	Yes
A581 (8.34)			No
T589 (8.54)	T440	5.35	Yes
S587 (8.72)	G438	16.24	Yes
A592 (8.83)	N443	5.50	Yes
Q486 (8.93) **	Y342	3.00	No
N497 (9.36) **			Yes
N496 (9.87) **			Yes
S490 (10.06) **			Yes
N498 (10.27) **			Yes
F534 (10.32) **	E368	4.30	Yes
P536 (10.37) **	Q370	2.20	Yes
R576 (10.59) **	G412	8.56	Yes
R485 (10.67) **	P341	3.82	Yes
G505 (10.69) **			No
M537 (11.08) **	A371	4.58	Yes
F535 (11.11) **	N369	4.35	Yes
T593 (11.14)	Y444	9.35	Yes
A573 (11.34) **	Y409	6.69	No
D495 (11.53) **			Yes
F501 (11.71) **			Yes
A506 (11.99) **	Y343	3.83	No
V580 (12.10)	Q416	6.66	Yes
H597 (12.27) **	F448	2.19	Yes
RMSD (surface-exposed amino acids only) = 7.37 Å			

Supplementary Table S10. Differences in topology around the AAV6 L584 and FPV I418 regions. *Distances from AAV6 L584 in Cα positions. **They are from neighboring subunits.
